# Protection Elicited by Attenuated Live *Yersinia pestis* Vaccine Strains against Lethal Infection with Virulent *Y. pestis*

**DOI:** 10.3390/vaccines9020161

**Published:** 2021-02-16

**Authors:** Christopher K. Cote, Sergei S. Biryukov, Christopher P. Klimko, Jennifer L. Shoe, Melissa Hunter, Raysa Rosario-Acevedo, David P. Fetterer, Krishna L. Moody, Joshua R. Meyer, Nathaniel O. Rill, Jennifer L. Dankmeyer, Patricia L. Worsham, Joel A. Bozue, Susan L. Welkos

**Affiliations:** Bacteriology Division, United States Army Medical Research Institute of Infectious Diseases (USAMRIID), Fort Detrick, MD 21702, USA; sergei.s.biryukov.mil@mail.mil (S.S.B.); christopher.p.klimko2.ctr@mail.mil (C.P.K.); jennifer.l.shoe.ctr@mail.mil (J.L.S.); melissa.hunter.ctr@mail.mil (M.H.); raysa.rosarioacevedo.mil@mail.mil (R.R.-A.); david.p.fetterer.ctr@mail.mil (D.P.F.); moody_krishna_laroche@lilly.com (K.L.M.); joshua.r.meyer15.mil@mail.mil (J.R.M.); nathaniel.r.rill.ctr@mail.mil (N.O.R.); Jennifer.l.dankmeyer.ctr@mail.mil (J.L.D.); patricia.l.worsham.civ@mail.mil (P.L.W.); joel.a.bozue.civ@mail.mil (J.A.B.); susan.l.welkos.vol@mail.mil (S.L.W.)

**Keywords:** plague, *Yersinia pestis*, vaccine, mice, bubonic, pneumonic, live attenuated vaccine, phage shock protein (PSP)

## Abstract

The etiologic agent of plague, *Yersinia pestis*, is a globally distributed pathogen which poses both a natural and adversarial threat. Due largely to the rapid course and high mortality of pneumonic plague, vaccines are greatly needed. Two-component protein vaccines have been unreliable and potentially vulnerable to vaccine resistance. We evaluated the safety and efficacy of eight live *Y. pestis* strains derived from virulent strains CO92 or KIM6+ and mutated in one or more virulence-associated gene(s) or cured of plasmid pPst. Stringent, single-dose vaccination allowed down-selection of the two safest and most protective vaccine candidates, CO92 mutants *pgm*- pPst- and Δ*yscN*. Both completely protected BALB/c mice against subcutaneous and aerosol challenge with *Y. pestis*. Strain CD-1 outbred mice were more resistant to bubonic (but not pneumonic) plague than BALB/c mice, but the vaccines elicited partial protection of CD-1 mice against aerosol challenge, while providing full protection against subcutaneous challenge. A Δ*yscN* mutant of the nonencapsulated C12 strain was expected to display antigens previously concealed by the capsule. C12 Δ*yscN* elicited negligible titers to F1 but comparable antibody levels to whole killed bacteria, as did CO92 Δ*yscN*. Although one dose of C12 Δ*yscN* was not protective, vaccination with two doses of either CO92 Δ*yscN,* or a combination of the Δysc*N* mutants of C12 and CO92, protected optimally against lethal bubonic or pneumonic plague. Protection against encapsulated *Y. pestis* required inclusion of F1 in the vaccine and was associated with high anti-F1 titers.

## 1. Introduction

The first plague vaccines were developed late in the 19th century and consisted of killed whole cells of *Yersinia pestis* [1]. Later, an immunogenic and less reactogenic vaccine which contained a formalin-killed suspension of virulent plague bacilli (Plague Vaccine U.S.P.; also known as the Cutter vaccine) was developed and licensed. It had been routinely given to military personnel stationed in Vietnam and other individuals, such as field personnel working in plague endemic areas with exposure to rats and fleas and laboratory personnel working with *Y. pestis* [2]. Although it was effective in preventing or ameliorating bubonic disease, as seen by the low incidence of plague in military personnel serving in Vietnam, animal data suggested that this vaccine might not protect against pneumonic plague [3,4]. Moreover, the only major protective antigen in these vaccines was the F1 capsular antigen. Such vaccines do not protect against genetically engineered or naturally occurring F1-negative strains, which often maintain virulence despite the loss of capsule [5,6,7]. A human plague vaccine candidate currently in clinical trials is F1-V, a fusion protein of F1 and LcrV, the low calcium response virulence protein (V), a key immunogen and anti-host factor, respectively. V is required for translocation of the immunomodulatory Yersinia outer proteins (Yops), effector proteins translocated by the type three secretion system (T3SS) into host cells, and it stimulates production of immunosuppressive cytokines [8]. The F1-V vaccine was shown to be efficacious in mice and some, but not all, nonhuman primate species [4,9,10,11,12,13,14]. Thus, a more efficacious plague vaccine that can induce an enhanced antibody and cell-mediated immune response in large animal models may be needed. Moreover, the protection afforded by F1-V against virulent F1-negative strains relies entirely on the V antigen component. Since there is evidence for V heterogeneity within *Yersinia* species [15,16,17,18], the potential exists that naturally occurring or engineered strains harboring altered V antigens could overcome F1-V induced immunity [4].

Other options for prophylactic protection against plague include using live attenuated strains. The former Soviet Union and other nations have traditionally focused on live attenuated vaccines, and millions of humans have received live plague vaccines [19,20]. Live bacterial plague vaccines offer several potential advantages. Live vaccines might provide better protection than subunit vaccines against virulent F1-negative or V-altered *Y. pestis* strains, due to their presentation of multiple antigens. Moreover, living strains have the potential capacity to induce both humoral and cellular immune responses. Whereas humoral immunity is often more prominent in subunit vaccines given with an adjuvant such as alhydrogel, live vaccines often can induce long-term protective immunity after a few doses [1,3,19]. Although the importance of antibody in plague immunity is well established, a number of studies also support the role of cellular immunity in protection against plague [1,4,19,21,22]. Animals immunized with live vaccine preparations have survived *Y. pestis* challenge with little measurable antibody titers, indicating that cellular immunity contributes to protective immunity [23,24,25,26,27]. Disadvantages of live vaccines include reactogenicity and residual pathogenicity [28,29]. Moreover, comparisons of the efficacy of *Y. pestis* live vaccines have been challenging, due in part to their often incompletely defined genetic composition. Extensive reviews of both recombinant and live plague vaccines are available [1,4,19,21].

In addition to live plague vaccines derived from *Y. pestis*, candidate vaccines have included recombinant and attenuated strains of *Salmonella*, *Yersinia pseudotuberculosis*, or other bacteria [1,3,30]. Most recently, *Y. pseudotuberculosis* strains modified to express the *Y. pestis* F1 capsule have been developed and evaluated [3,31,32,33,34]. Although *Y. pseudotuberculosis* is genetically very similar to *Y. pestis*, only vaccines derived from *Y. pestis* would be assured to possess the full component of genetically identical antigens. The *Y. pseudotuberculosis* vaccines also do not produce the pPCP1 (pPst) and pMT1 (pFra) plasmid encoded proteins and virulence factors, such as plasminogen activator (*pla*) and mouse toxin phospholipase D (*ymt*); and some *Y. pseudotuberculosis* strains exhibit differences from *Y. pestis* in their T3SS and encoded effector proteins [35]. In addition, *Y. pseudotuberculosis* vaccines are often presented to animals by the intragastric route, which introduces a possibly greater risk (compared to a parental route) of an inaccurate or harmful delivery.

Our objective is to develop next generation live vaccines which address the potential threat of emerging and genetically engineered strains of *Y. pestis*. Initial efforts have been focused on confirming and optimizing some of the most promising existing vaccine candidates for safety, immunogenicity, and efficacy. We have tested a panel of *Y. pestis* vaccine strains for the down-selection of a potential candidate vaccine(s) in mouse models of bubonic and pneumonic plague.

## 2. Materials and Methods

### 2.1. Media and Chemicals

The *Y. pestis* CO92 mutant strains were grown in heart infusion broth (HIB) medium supplemented with 0.2% xylose (HIBX). KIM6+ χ10030/pCD1Ap1 strain was grown in HIB supplemented with 0.1% arabinose and 50 µg/mL ampicillin. For all strains, animal vaccines were prepared with cultures incubated in HIBX supplemented with 2.5 mM CaCl_2_. The *Y. pestis* CO92 mutant strains were plated on 5% sheep blood agar plates (SBAP) or tryptose blood agar base slants, and the KIM6+ χ10030/pCD1Ap1 strain was plated on Congo Red agar with 0.1% arabinose and 100 µg/mL ampicillin. A solution of 10 mM potassium phosphate, pH 7.3–7.4 (Kphos), was used as the buffer diluent [27]. Bacteriological media were from Thermo Fisher-Remel and chemicals from Sigma-Aldrich (St. Louis, MO, USA).

### 2.2. Mutant Construction

The *yscN* deletion was constructed in the C12 *Y. pestis* background, an F1-negative capsule minus strain [7], and confirmed as described previously [27]. For constructing the Δ*pspA* and Δ*pspC* mutants in the *Y. pestis* CO92 strain, a fragment of DNA containing the respective gene and overlapping sequence was PCR amplified from genomic DNA and primers (Table 1).

The PCR product containing the *pspA* or *pspC* open reading frame and flanking DNA sequences was ligated into plasmid vector pWKS30 and then removed through inverse PCR. The *Y. pestis* DNA containing the respective deletion was then subcloned into pCVD422 [36]. Construction of the *Y. pestis* mutants were performed as previously described [37]. The deletions were screened and shown to be correct by PCR analyses using the primers listed in Table 1. The presence of all *Y. pestis* virulence plasmids was confirmed via PCR amplification.

### 2.3. LD_50_ Determinations

As indicated in Table 2, LD_50_ determinations with some of the *Y. pestis* CO92 mutant strains making up this panel were initially performed with Swiss Webster and/or BALB/c mice (6–8 weeks old) in groups of 10 by subcutaneous (SC), intranasal instillation, or whole body aerosol challenge, as previously described [38]. Additionally, historical data exist for nearly all of the strains used in Swiss Webster mice [6,7,8,39,40].

For all methods of infection, the challenge doses were determined by serial dilutions in Kphos buffer and plating on sheep blood agar.

### 2.4. Bacterial Strains

The wild type virulent *Y. pestis* CO92, and its F1-negative (nonencapsulated) derivative, C12, were used [7,41]. The derived mutant strains shown in Table 2 were available from collections at the USAMRIID. These strains had been constructed and characterized as described previously, and they had mutations in virulence-associated genes, i.e., *yscN*, *pspA*, *pspC,* and *tatA*, or deletion of the *pgm* locus and curing of the pPst plasmid [27,38,42,43,44]. The mutant strain *Y. pestis* KIM6+ χ10030 was kindly provided by Dr. R. Curtiss (U. Florida, Gainesville, FL, USA) and Dr. W. Sun (Albany Medical College, Albany, NY, USA). Strain KIM6+ χ10030 was stably electrotransformed with plasmid pCD1Ap1 DNA (obtained from R. Curtiss and W. Sun) under BSL-3 conditions [45,46,47]. It is an ampicillin resistance-marked derivative of the pCD1 (pLcr) virulence plasmid. The χ10030/pCD1Ap1 transformants were isolated on Congo Red agar supplemented with ampicillin and incubated at 28–30 °C. The ampicillin resistant transformants formed red colonies, and thus, the transformed strain carried the genetic material for the *pgm* locus and the *lcrV* gene. Although χ10030/pCD1Ap1 carries the genes for all potential virulence factors and vaccine antigens, it is highly attenuated, due to altered in vivo expression of a global regulatory gene (*crp*), which is under transcriptional control of the *araC* pBAD promoter, and to the production of an immune-stimulatory form of lipidA (*lpxL*), as detailed previously [3,47,48,49].

### 2.5. Animals and Vaccination Studies

The animal research was conducted under an animal use protocol approved by the USAMRIID Institutional Animal Care and Use Committee (IACUC) in compliance with the Animal Welfare Act, PHS Policy, and other Federal statutes and regulations relating to animals and experiments involving animals. The facility where this research was conducted is accredited by the AAALAC International and adheres to principles stated in the Guide for the Care and Use of Laboratory Animals (National Research Council, 2011). Mice were obtained from Charles River (Frederick, MD, USA) and included females of the inbred BALB/c and random bred CD-1 strains that were 7–10 weeks of age at time of vaccination. Except as indicated, one dose of vaccine was administered via subcutaneous (SC) injection and the mice exposed four weeks later by the aerosol or SC route to a lethal dose of *Y. pestis* CO92; mice vaccinated twice were administered the second dose 21–28 days after the initial vaccine dose. Sera and spleens were collected from a cohort of mice to assess immune responses to the vaccines. Mice were challenged 28–30 days post final vaccination.

### 2.6. Preparation of Vaccine Strains for Immunizations

On the day before vaccination, flasks were inoculated with a suspension of colonies from a freshly inoculated agar plate and the broths incubated for 24 h at 28–30 °C with shaking at 200 rpm. On the next day, the cultures were adjusted to an OD_600_ of 0.1 in fresh medium and incubated to the OD_600_ determined to produce the target CFU concentration, which was 10^7^ CFU in doses of 0.2 mL (as recommended by R. Curtiss). To confirm the actual delivered dose of bacteria, the final suspensions were diluted and plated for viable counts. All plates were incubated at 28–30 °C for two days before counting.

### 2.7. Exposure of Immunized Mice to Virulent Y. pestis Challenge

Mice were exposed to aerosolized (pneumonic) or SC (bubonic) challenge doses of virulent *Y. pestis* that were prepared as previously described [27,38,50]. For bubonic plague challenge, bacteria were harvested from tryptose blood agar (TBA) slants. Mice exposed by the SC route were inoculated with 0.2 mL volumes of the suspension in Kphos [27,38]. The bacteria used for aerosol studies were prepared by using colonies from freshly inoculated TBA slants which were suspended in HIBX and incubated for approximately 24 h at 28–30°C. For pneumonic plague challenge, the cultures were harvested by centrifugation and suspended in HIB medium (no xylose) to the concentration yielding the number of LD_50_ doses indicated in the tables. Exposure to aerosolized bacteria was accomplished as previously described [27,38,51]. Briefly, mice were transferred to wire mesh cages and were placed in a whole-body aerosol chamber within a class three biological safety cabinet located inside a BSL-3 laboratory. Mice were exposed to aerosols of *Y. pestis* strain CO92 created by a three-jet collison nebulizer. Samples were collected from the all-glass impinger (AGI) vessel and analyzed by performing CFU calculations to determine the inhaled dose of *Y. pestis.*

### 2.8. Immune Response Assays

#### 2.8.1. ELISAs

Immunoglobulin (Ig) IgG, IgG1, and IgG2a antibody responses to the live vaccines were determined by semi-quantitative endpoint ELISA using sera from vaccinated BALB/c mice, as previously described [46]. The sera were collected as terminal blood collections from axillary vessels and titrated against several capture antigens: F1 protein, V protein, the F1-V recombinant fusion protein, and γ-radiation inactivated whole cells of *Y. pestis* strains CO92 and C12 either grown at 30 °C (24 h), or 30 °C for 21 h followed by a switch to 37 °C and incubation for an additional three h to upregulate the presentation of potential antigens. The F1 and V antigens (BEI resources; Manassas, VA, USA) and the F1-V fusion protein vaccine construct were diluted in 0.1 M carbonate buffer, pH 9.5, to a concentration of 2 μg/mL, while inactivated *Y. pestis* CO92 or C12 whole cells were plated at a concentration of 10 μg/mL on 96-well Immulon 2HB plates (ThermoFisher, Grand Island, NY, USA). Plates were stored at 4 °C overnight, then washed and blocked, and samples were processed as previously described (8). Two-fold dilutions of the serum were made in triplicate and the results are reported as the geometric mean (Geo Mean) and geometric standard error (GSE) of the reciprocal of the highest dilution giving a mean OD of at least 0.1 ± 1 SD at 450 nm with a reference filter (570 nm). Samples with an antibody titer of <50 were considered negative.

#### 2.8.2. Cellular Responses: Analysis of Stimulated Splenocytes

BALB/c mice vaccinated with live *Yersinia* mutant strains were necropsied to excise spleens 28–30 days after the primary vaccine or the booster vaccine dose, depending upon the vaccine regimen being tested. The mice used for these analyses received their booster vaccine 28 days after the primary vaccination. Splenocytes were extracted through manual disruption in RPMI 1640 (ThermoFisher, Grand Island, NY, USA) 60 mm petri dishes, large debris was allowed to settle and the supernatant was transferred to a fresh conical tube. Splenocytes were diluted to 15 mL with additional RPMI 1640 and spun at 1200 rpm (335× *g*) for 10 min at room temperature (RT). The supernatant was discarded and the pellet was resuspended in 4 mL ACK Lysis buffer (Lonza, Walkersville, MD, USA), incubated for 5 min at RT, then 10 mL of RPMI 1640 was added to stop the reaction. Samples were rested for 5 min at RT to allow debris to settle and the supernatant was carefully decanted into a fresh 15 mL tube for centrifugation at 1200 rpm (335 × *g*) for 10 min, RT. The supernatant was discarded, the pellet resuspended in RPMI 1640 complete media (10% FBS, etc.), and the cells counted with a TC20 Cell Counter (BioRad). Splenocytes were diluted to a 1 × 10^7^/mL concentration in RPMI complete medium and stimulated in vitro with rF1-V (25 µg/mL) protein, γ-radiation inactivated *Y. pestis* CO92 (5 µg/mL) or temperature-shifted *Y. pestis* C12 (5 µg/mL) bacteria and incubated at 37 °C with 5% CO_2_ for approximately 48 h. Plates were then centrifuged for 1200× *g* (for 10 min at RT) and the supernatants collected for evaluation of cytokine expression by Luminex Mag Pix 36-plex mouse panel per manufacturer directions (Thermo Fisher Scientific, Grand Island, NY, USA). Splenocytes from uninfected BALB/c mice were used as normal, uninfected controls; and stimulations with medium alone or 100 ng/mL PMA/0.5 μg/mL Ionomycin (Sigma Aldrich, St. Louis, MO, USA) were used as negative and positive controls for stimulation, respectively. The levels (pg/mL) of the following cytokines/chemokines were measured: Eotaxin, ENA-78/CXCL5, G-CSF, GM-CSF, IFN-γ, IL-1α, IL-1β, IL-2, IL-4, IL-5, IL-6, IL-9, IL-10, IL-12 (p70), IL-13, IL-15/IL-15R, IL-17A, IL-18, IL-22, IL-23, IL-27, IL-28; IL-31, IP-10, LIF, M-CSF, MCP-3, MIG, MIP-1α, MIP-1β, MIP-2, RANTES, and TNF-α. Only cytokines that exhibited elevated levels at least 4-fold higher than normal, uninfected controls were reported.

### 2.9. Statistics

For Luminex analysis, the splenocyte samples were tested in duplicate with replicates of 2–3 each, for a total of 4–5 values per sample. The geometric mean and geometric standard error were determined for each group, and the data were evaluated by applying ANOVA to the log transformed values. The results from the vaccinated groups were compared to that of the unvaccinated Kphos buffer control group; statistically significant comparisons were those with *p* < 0.05. For each cytokine, the vaccinated group samples were normalized by determining the fold change compared to the buffer control mice results using the geometric mean data (pg/mL). The graphs were prepared using GraphPad Prism version 8.0.0 software for Windows (GraphPad Software, San Diego, CA, USA). ELISA titers and IgG2a/IgG1 ratios were log transformed prior to analysis and compared by Welch’s *t*-test. Results were summarized as geometric mean (Geo Mean) titer and geometric standard error (GSE).

## 3. Results

### 3.1. Characterization of Virulent and Live Attenuated Y. pestis Strains

We prepared various attenuated mutant strains of the wild type parent CO92 strain of *Y. pestis* in efforts to discern factors required for full virulence of *Y. pestis*, to develop surrogate strains for use in lower biosafety level containment laboratories, and to generate potential live vaccine strains. Some of these mutant strains have been previously described, such as the 102 kB deletion of the pigmentation locus (*pgm*) [2,3,52,53,54], the twin arginine translocation pathway gene *tatA* [38], the *yscN* gene-encoded ATPase [27,43], and the entire 9.5-kb pPst plasmid (also designated pPCP1, pPla, or pY. PESTIS) encoding the plasminogen activator protease, the pesticin bacteriocin, and a pesticin immunity protein (Table 2) [55]. In addition, a Δ*yscN* mutant derived from the virulent *Y. pestis* strain C12 was created and used in this current study [27]. Strain C12 is a derivative of CO92 harboring a stop codon at the initiation of the *caf1A* gene of the *caf1* operon which prevents synthesis of the fraction 1 (F1) capsule protein [7]. Finally, we also acquired a live vaccine candidate strain, χ10030/pCD1Ap1, kindly provided by Dr. R Curtiss and Dr. W. Sun, which was derived from the virulent KIM6+ strain of *Y. pestis*. This vaccine candidate carries the arabinose-regulated *crp* gene and expresses the TLR4-reactive *E. coli lpxL* (hexa-acylated lipid A) LPS variant [3]. The strain was electrotransformed with the plasmid derivative of pCD1 (pLcr), as described above, to allow presentation of the TTSS proteins including the V protein virulence factor.

In addition to these previously described mutants, here we also tested novel strains derived from CO92 with mutations in the genes encoding for the *Y. pestis* phage shock proteins, *pspA* and *pspC*. The phage shock protein (PSP) system is a stress response to the cell envelope and has been studied extensively in *E. coli* and shown to be essential for virulence in *Yersinia enterolitica* [56,57]. We demonstrated that PspA (a proposed regulatory protein) and PspC (a polytopic membrane protein) are also important for the pathogenesis of *Y. pestis* after either bubonic or aerosol challenge (Table 2).

The CO92 in-frame deletion of *pspA* (Δ*pspA*) was shown to be highly attenuated for bubonic challenge (LD_50_ of 600 CFU) versus the LD_50_ of 1–2 CFU for the parent strain. When mice were challenged by whole body aerosol with Δ*pspA*, no LD_50_ dose could be reached as 5/10 of the highest challenged group survived (LD_50_ > 1 × 10^6^ CFU) (Table 2 and Supplementary Figure S1). In contrast, the aerosol LD_50_ for the parent CO92 strain is 6.8 × 10^4^ CFU [50].

The *Y. pestis psp* locus appears to be in an operon similarly as in *Y. enterocolitica* and *E. coli* [56]. For this study, we analyzed two mutated versions of *pspC*. The initial CO92 *pspC* mutant had a frameshift mutation in the gene, preventing its expression, and it was referred to as Δ*pspC*I. In the second *pspC* mutant (Δ*pspC*II), the complete gene sequence was deleted in-frame to ensure transcription of the downstream genes were unaffected. For LD_50_ measurements by SC challenge, both mutants showed a high level of attenuation. However, for the *pspC*I mutant, we were unable to calculate a statistically robust LD_50_ value because only 6/10 mice succumbed to the highest challenge dose (LD_50_ > 2.14 × 10^5^ CFU). In contrast, the LD_50_ for the in-frame Δ*pspC*II mutant was determined to be approximately 2 logs lower (3.1 × 10^3^ CFU). When examining the role of the *pspC* gene in pneumonic plague, both versions of the *pspC* mutants were found to be highly attenuated by whole body aerosol challenge with the LD_50_ being greater than 10^6^ CFU because 50% or more of the mice survived challenge in the highest exposed groups (Table 2 and Supplementary Figure S1).

Table 2 summarizes the reported lethality for mice of all the mutants evaluated in this study. For live vaccine efficacy tests in mice, we selected a target vaccine dose of approximately 1 × 10^7^ CFU ([47], R. Curtiss personal communication). This vaccine dose was found to be greater than the LD_50_ of some of the mutants, and we confirmed that several of the mutant candidates retained an unacceptable degree of virulence.

### 3.2. Safety and Efficacy of Live Vaccine Strain Candidates

The safety of the eight live vaccine candidates (Table 3) was evaluated in BALB/c mice exposed by the SC route.

Four of the CO92 derivatives harboring a single attenuating mutation were significantly attenuated but were lethal at the selected vaccination dose (Table 2 and Table 3). Despite its extensive attenuation in Swiss Webster mice [3,47], the KIM6+ strain χ10030/pCD1Ap retained significant virulence in BALB/c mice administered doses, which were 14- to 24-fold less than the targeted 10^7^ CFU dose. These five strains were subsequently removed from consideration, and the three safest ones retained for further characterization: CO92 *pgm*-pPst-, a double mutant with a deletion of the entire *pgm* locus and cured of plasmid pPst; CO92 with an inactivation of the *yscN* gene; and the F1-negative strain C12 with an inactivation of the *yscN* gene. These three selected strains were evaluated for protection against challenge by the virulent *Y. pestis* strain CO92.

Our initial vaccine down-selection strategy utilized stringent conditions to rapidly identify the safest and most protective attenuated strain. A single dose of live vaccine was administered to BALB/c mice which are highly susceptible to infection with *Y. pestis*. The safety and efficacy of the vaccines after exposure to a lethal dose of wild *Y. pestis* CO92 are shown in Table 3. Three of the vaccine strains were again nonlethal at doses of approximately 1 × 10^7^ CFU, and two protected mice completely against lethal exposure by the SC and aerosol routes to *Y. pestis* strain CO92. Mice vaccinated with a single dose of C12 Δ*yscN* were only partially protected (30%) against virulent SC challenge and were not protected from exposure to aerosolized CO92 in this experiment.

### 3.3. Characterization of the Outbred CD-1 Mouse Strain: Susceptibility and Vaccine Responses

The outbred CD-1 mouse strain was assessed as an alternate and potentially less susceptible murine host. To standardize the doses of the virulent challenge strains administered to both strains of mice, the SC and aerosol LD_50_ values were determined as described previously [27,51]. *Y. pestis* CO92 had a SC LD_50_ for CD-1 mice that was approximately 30-fold higher than that for BALB/c (52 vs. 1-2 CFU, respectively). However, the aerosol LD_50_ estimates were similar and most likely not statistically significant (3.4 × 10^4^ compared to 6.8 × 10^4^ [50], respectively, for CD-1 and BALB/c mice). Accordingly, CD-1 mice represent a more resistant model of bubonic plague. The vaccine strains CO92 *pgm*-pPst- and CO92 Δ*yscN* were completely attenuated at all doses tested for CD-1 mice, producing no mortalities. As shown in Table 4, one dose of either vaccine strain was 100% protective for CD-1 mice against SC challenge with CO92.

The vaccines tested elicited only partial protection of CD-1 mice against a lethal aerosolized challenge dose of CO92. Survival rates were 60% for strain CO92 *pgm*-pPst-vaccinated mice and 20% for strain CO92 Δ*yscN* vaccinated mice. Although the amount of aerosolized bacteria delivered was considerably higher in the CD-1 challenge experiment compared to the BALB/c experiment, these aerosol survival data are in contrast to the full protection induced by the two vaccine strains in BALB/c mice.

### 3.4. Humoral Immune Responses Elicited by Vaccines

Sera were collected from vaccinated mice 4 weeks post-vaccination and assayed by ELISA for antibody titers against three antigens, killed CO92 and C12 whole cells and the Fl-V recombinant fusion protein plague vaccine. The sera were obtained from mice vaccinated with one of the three vaccine strains; an additional group had been vaccinated with a combination of equal numbers of both the CO92 and C12 Δ*yscN* mutants (Combo). As illustrated by the mean titer data in Table 5, all vaccines containing a CO92-derived strain (the *pgm*- pPst- mutant or Δ*yscN* mutant) elicited high titers to F1-V, whereas the Δ*yscN* mutant of the F1^-^ C12 strain by itself stimulated a negligible anti-F1-V titer (*p* < 0.0001).

In addition, the mice receiving the *pgm*-pPst-vaccine produced significantly higher anti-F1-V titers compared to the CO92 Δ*yscN* or Combo (CO92 Δ*yscN* + C12 Δ*yscN*) vaccines (*p* = 0.043 and *p* = 0.031, respectively).

A single dose of all of the vaccines also elicited antibody responses to the two whole bacterial antigen preparations, but the titers were several folds less than the titers to F1-V (Table 5). The CO92 *pgm*- pPst- strain induced the highest titer to the killed bacteria compared to CO92 Δ*yscN* (*p* < 0.0014 for either killed whole-cell antigen) or Combo (*p* = 0.016 when killed CO92 was the ELISA antigen). The mice receiving the C12 Δ*yscN* vaccine produced higher antibody titers against the killed antigen preparations compared to mice receiving the CO92 Δ*yscN* vaccine (*p* < 0.025 for either antigen preparation). As the CO92 Δ*yscN-*elicited anti-whole cell titers that were negligible, we presume that this was due primarily to the prevalence of the anti-F1 immune response that is absent in the mice receiving the C12 Δ*yscN* vaccine or the fact that the capsule could be masking the presentation of the other non-capsular antigens to the vaccinees (Table 5).

### 3.5. Vaccine Optimization: Comparison of Vaccine Composition and Number of Doses

#### 3.5.1. Protective Efficacy

To improve protection afforded by two of the live vaccine strains, we evaluated the efficacy of the two Δ*yscN* mutants (CO92 or C12) alone or in combination, with one or two doses. Groups of mice were administered vaccine or Kphos SC and then exposed to *Y. pestis* CO92 by the SC or aerosol routes (Table 6).

Optimal protection against lethal infection by both routes was achieved by vaccination with two doses of either the CO92 Δ*yscN* mutant, alone or a combination of the Δ*yscN* mutants of the C12 and CO92 strains (90–100% survival, compared to no survivors in the Kphos control group). One or two doses of the vaccines containing only the C12 Δ*yscN* mutant were not effective; survival rates ranged from 0% to 40%. Thus, protection against CO92 required the presence of an F1 capsule-producing strain in the vaccine.

#### 3.5.2. Humoral Immune Responses

The serum antibody responses to four antigens (F1 and V recombinant proteins and killed CO92 and C12 whole bacteria) were determined. ELISA data on pre-challenge sera from the mice are shown in Table 7.

The mice vaccinated with a single dose of CO92 *pgm*-pPst-vaccine produced significantly higher titers against the F1 and V antigens compared to all other vaccines delivered as a single dose regimen (*p* < 0.002 in all comparisons). While statistically significant, the biological relevance of the difference in anti-V titers is unclear because of the overall low titers achieved. All of the vaccines containing a CO92-derived mutant induced moderate to high IgG responses to F1 capsule, and the response appeared to be vaccine boost-related. As expected, the mice receiving a single dose of CO92 Δ*yscN* had higher F1 antibodies compared to mice receiving a single dose of C12 Δ*yscN* or Combo (CO92 Δ*yscN* + C12 Δ*yscN*) (*p* < 0.001 and *p* = 0.17, respectively). These differences were only noted for the double dose vaccine when comparing CO92 Δ*yscN* or Combo with C12 Δ*yscN* (*p* < 0.0001). A double dose of *pgm*- pPst- significantly increased the anti-F1 titer compared to a single dose (*p* = 0.0005). However, the second dose of the *pgm*- pPst- vaccine did not increase anti-V titers to a statistically significant level. These data support the efficacy results (Table 6) and the implied importance of anti-F1 antibody in protection against CO92 challenge. None of the ∆*yscN* vaccines elicited significant anti-V antibody responses, as might be expected due to the requirement of the YscN ATPase for a functional T3SS (Table 7). However, the absence of a robust anti-V response in animals or humans vaccinated with live attenuated *Y. pestis* strains has been previously reported [32,58,59,60,61,62,63]. A slight increase in anti-V titers was observed in mice receiving two doses of CO92 Δ*yscN* as compared to mice receiving two doses of C12 Δ*yscN* (*p* = 0.034).

Finally, we prepared whole cell killed cell antigens from cultures that were grown at 30 °C (Table 8) or were subjected to a temperature switch from 30 °C to 37 °C (Table 9).

This temperature switch allowed us to assess the immune response against a more robust capsule and other temperature inducible antigens (e.g., V antigen or other T3SS structural or secreted proteins). Booster doses of all three *ΔyscN*-containing vaccines yielded significant increases in antibodies to both whole bacterial antigens compared to a single vaccine dose (*p* < 0.002). The titers against killed whole-cell CO92 (Table 8 and Table 9) were considerably lower than the titers determined against the F1 protein (Table 7).

As expected, the titers generated using these antigen preparations revealed a higher antibody response against the organisms that were exposed to 37 °C growth conditions (Table 9). When the vaccine strains are compared as single dose regimen, the mice receiving the CO92 *pgm*-pPst-vaccine had significantly higher total IgG (*p* < 0.014) and IgG1 (*p* < 0.012) levels against either CO92 or C12 killed whole cell antigen preparations (regardless of the temperature used to grow the bacteria used for the antigen preparations) compared to all other single dose vaccine regimens (Table 9). When mice received two doses of the CO92 *pgm*-pPst-vaccine, the titers in all parameters tested were significantly higher than those determined in mice receiving a single dose of that vaccine (*p* < 0.009). There was also a significant difference in the IgG2a titers for the mice receiving the CO92 *pgm*- pPst- vaccine compared to mice receiving the C12 ∆*yscN* vaccine (*p* < 0.02). Mice receiving the CO92 ∆*yscN* vaccine strain only or the Combo vaccine strains had significantly higher total IgG titers against killed CO92 whole cell antigen preparations compared to the C12 ∆*yscN* vaccine group (*p* = 0.001 and 0.008, respectively). The same observations were noted when comparing IgG1 levels against temperature switched CO92 generated by mice receiving the CO92 ∆*yscN* vaccine strain or the Combo vaccine compared to mice receiving the C12 ∆*yscN* vaccine (*p* = 0.0006 and *p* = 0.0044, respectively). This trend continued when analyzing IgG2a titers, but statistical significance was only achieved when comparing the anti-CO92 titers generated in the mice receiving the CO92 ∆*yscN* vaccine strain compared to mice receiving the C12 ∆*yscN* (*p* = 0.017). These differences can likely be attributed to the immune response to the F1 antigen produced in both vaccines containing an attenuated CO92 vaccine strain. Production of IgG1 antibodies correlates with an overall Th2-like immune response profile while that of IgG2a antibodies are indicative of an overall Th1-like profile. A higher IgG2a/IgG1 ratio would be suggestive of an enhancement of a Th1 response. Unfortunately, no pronounced induction of IgG2a was observed in any of the vaccine groups relative to IgG1, with the exception of a >3-fold increase in IgG2a/IgG1 ratio between the single and the double CO92 *pgm*-pPst-vaccine groups. Of note, this enhancement is only observed with the temperature shifted CO92 capture antigen. Furthermore, due to exceptionally low titers in some of the vaccine groups, some of the IgG2a/IgG1 ratios are artifactually inflated, such as in the anti-C12 titers from the Combo (CO92 Δ*yscN* + C12 Δ*yscN*) vaccinated group.

It was interesting to note that the mice receiving a double dose of the Combo vaccine demonstrated a trend of increased titers against whole cell antigen preparations compared to mice receiving either of the component live attenuated vaccines alone. This was observed even though the total number of CFU for each vaccine dose was approximately 1 × 10^7^. However, only a comparison of total IgG titers generated against killed CO92 (temperature-switched antigen), IgG1 titers generated against killed C12 (30 °C antigen), and IgG1 titers against killed CO92 (temperature-switched antigen) by mice vaccinated with the Combo vaccine compared to mice receiving the CO92 ∆*yscN* reached statistical significance (*p* < 0.042 for these comparisons) (Table 9).

#### 3.5.3. Cell-Mediated Immune Responses to the Live Vaccines

Splenocytes from the vaccinated mice (Table 6) were stimulated in vitro with F1-V or inactivated *Y. pestis* CO92 or C12 bacteria and the supernatants evaluated for cytokine expression by Luminex bead-based assays. In addition, groups receiving a single or double dose of *pgm*-pPst-vaccine were also included for immunological comparisons. Control samples included cells stimulated with medium alone, for background cytokine levels, or with PMA/ionomycin to confirm that the cells could be stimulated and were capable of producing a response (data not shown). Of the 36 cytokines tested, cytokines with at least a 4-fold increase in a vaccinated group over the control group (Kphos) are shown for each stimulation.

**Figure 1:**
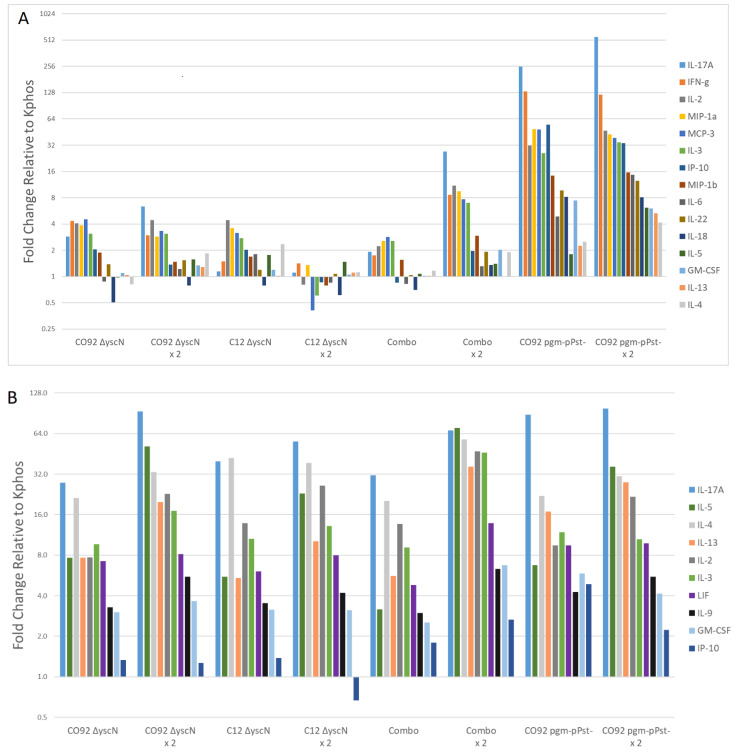

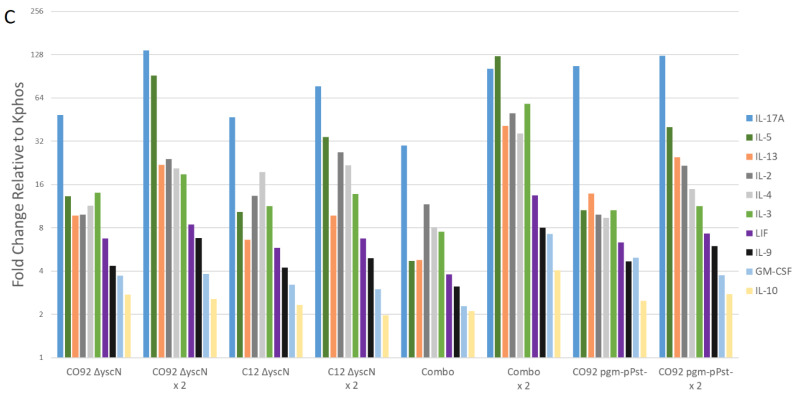
Splenocytes were harvested and re-stimulated for cytokine/chemokine expression with (**A**) F1-V fusion protein vaccine, (**B**) γ-radiation inactivated *Y. pestis* CO92, or (**C**) γ-radiation inactivated *Y. pestis* C12 (nonencapsulated) bacteria and the supernatants were evaluated for cytokine expression by Luminex (*n* = 5 for each group). The fold changes in cytokines/chemokines in splenocytes were determined by dividing the geometric mean of log-transformed data (pg/mL) of the cytokine/chemokine by that of the negative control (Kphos) within each group of vaccinated mice. Of note, the antigen used to stimulate the splenocytes resulted in differential cytokine expression profiles (e.g., IFN-γ was significantly upregulated by the addition of the F1-V immunogen but was not significantly upregulated by either killed whole cell antigen relative to naïve mice). Due to the less defined whole bacterial cell immunogens and their impacts on splenocytes harvested from naïve mice, the fold change data normalize the immune responses elicited by the F1-V recombinant protein compared to γ-irradiated *Y. pestis* cells. The Y-axis (fold change relative to Kphos) scale is different in each graph.

Groups vaccinated with a single or double dose of CO92 *pgm*-pPst-produced an overall greater cytokine response relative to groups vaccinated with Δ*yscN* mutants when stimulated with F1-V. Furthermore, Combo ×2 and CO92 *pgm*-pPst- ×2 vaccine regimens also induced higher expression overall of the majority of reported cytokines relative to their single dose counterparts (Figure 1A).

The levels of IL-17A, IFN-γ, IL-2, MIP-1a, MCP-3, IL-3, IP-10, MIP-1b, IL-22, and IL-18 were significantly higher in both CO92 *pgm*-pPst-vaccinated groups relative to groups vaccinated with Δ*yscN* mutants (statistical significance ranging from *p* < 0.032 to *p* < 0.0001 in these comparisons). However, the differences between the two CO92 *pgm*-pPst-groups (single or double dose regimens) did not reach significance for these cytokines (Figure 1A). The fold change in IL-5 and IL-6 expression levels were significantly higher in CO92 *pgm*-pPst- ×2 vaccinated mice relative to all other vaccine groups (*p* < 0.032). The level of IL-13 was significantly higher (*p* ≤ 0.027) relative to other vaccine groups, with the exception of the single dose CO92 *pgm*-pPst-group. Moreover, the levels of GM-CSF were also significantly elevated (*p* ≤ 0.019) in both CO92 *pgm*-pPst-vaccinated groups relative to other vaccine groups with the exception of CO92 *pgm*-pPst- ×2 vaccine which did not reach a significantly greater expression level relative to its single dose CO92 *pgm*-pPst-counterpart or the Combo ×2. The level of IL-4 was higher in both CO92 *pgm*-pPst-vaccinated groups relative to other vaccine groups, but significance was only reached relative to CO92 Δ*yscN,* Combo x1, and C12 Δ*yscN* ×2 (*p* ≤ 0.046).

All double dose vaccine groups produced a stronger overall cytokine response relative to single dose vaccine groups stimulated with whole cell stimulants, CO92 or C12 (Figure 1B,C). Furthermore, in contrast to stimulation with F1-V, stimulation with killed whole cell CO92 or C12 preparations resulted in generation by the Combo ×2 vaccinated mouse group of the most pronounced cytokine response, eclipsing even the CO92 *pgm*- pPst- vaccinated groups (Figure 1A–C). Overall, IL-17A was the most upregulated cytokine under all three stimulation conditions. With the exception of C12 ∆*yscN* vaccinated mice stimulated with F1-V, IL-17A levels in all double dose vaccine groups trended higher than single dose vaccine groups, but statistical significance was only reached with Combo ×2 (statistical significance ranging from *p* < 0.007 to *p* < 0.0001 in these comparisons). Levels of IL-2 and IL-3 in the Combo ×2 vaccinated group were significantly (*p* < 0.023) higher relative to all other vaccine groups after CO92 stimulation, while the levels of IL-3 and IL-4 were significantly (*p* < 0.031) higher after C12 stimulation. Stimulation with CO92 or C12 cells also appeared to induce the highest levels of IL-5, IL-13, and IL-9 in the Combo ×2 vaccinated group, but the levels did not reach statistical significance relative to CO92 *pgm*-pPst- ×2 or CO92 Δ*yscN* ×2 (Figure 1B–C). Furthermore, the level of IL-10 in the Combo ×2 vaccinated group after C12 stimulation was significantly higher than that of all other groups, except for the CO92 *pgm*-pPst- ×2, CO92 Δ*yscN*, and CO92 Δ*yscN* ×2 vaccinated groups (Figure 1C).

## 4. Discussion

Plague vaccines based on attenuated live strains are theoretically advantageous compared to subunit vaccines (i.e., containing F1 and V), since they can potentially elicit immunity against numerous antigens, and thus, lessen the chance that a virulent strain refractory to such vaccines could be engineered by adversaries. The major findings of this study are summarized as follows:

1. We demonstrated an important role of the PSP response for *Y. pestis* virulence in both bubonic and pneumonic models of plague but did not down-select these mutant strains as potential live attenuated strains due to residual virulence.

2. Three of the vaccine strains were significantly attenuated, and one dose of either of the two F1-producing CO92 mutants (Δ*yscN* or *pgm*-pPst-strains) protected BALB/c mice fully against lethal exposure by SC or inhalational routes to *Y. pestis* CO92. Two vaccines (CO92 Δ*yscN*, alone or combined with C12 Δ*yscN*) were down-selected for further challenge investigations. These novel strains do not secrete the V antigen due to the disrupted T3SS and in the case of the C12 derived vaccine strain produces no F1. Thus, they allowed us to investigate protection afforded by potentially novel presented antigens.

3. The CD-1 outbred mouse strain was more resistant to bubonic plague, but similarly susceptible to pneumonic plague when compared to BALB/c mice. The CD-1 mice were less well protected than BALB/c inbred mice against aerosol challenge. These results might be attributable to differences in Th2 responses and/or possibly differences in mucosal humoral immunity between the two mouse strains [64,65]. It is also important to note that the CD-1 mice did inhale a greater number of aerosolized *Y. pestis* CFUs.

4. Full protection against CO92 required induction of an immune response to F1. The F1-negative C12 Δ*yscN* strain was poorly protective and failed to induce antibody responses to F1 or V. This vaccine strain stimulated antibodies to whole cell antigens, but a role for the latter was not conclusively implicated in protection against encapsulated *Y. pestis* CO92. In addition, it is likely that the presence of a robust capsule present on CO92 *pgm*-pPst-and CO92 Δ*yscN* could have also potentiated longer vaccine strain survival times, allowing for more replications of these live attenuated vaccine strains and the generation of a host immune response, as compared to the C12 Δ*yscN* vaccine strain. Accordingly, the capsule production in live attenuated vaccine strains can be important in several respects.

5. Cell-mediated immunity (CMI) responses, involving Th17 and Th2 cells, may contribute to the vaccine-induced protection, as shown by results of the cytokine analysis with whole cell stimulated splenocytes. The cytokine IL-17A, followed by IL-5, IL-4, IL-13, and IL-2, were the most highly stimulated cytokines in cells from animals vaccinated twice with three protective capsule-producing vaccine strains, CO92 *pgm*-pPst-, CO92 Δ*yscN* alone or in combination with C12 Δ*yscN*.

Several candidate strains were evaluated for use as live vaccines. Three of the strains were non-lethal for mice at doses >10^7^ CFU. The significant attenuation of these strains was largely, due to inactivation of the *yscN* gene or the deletion of the chromosomal 102 kB pigmentation locus (*pgm*) together with curing of pPst. The *yscN* mutation is highly attenuating, since it encodes the ATPase required by the *Yersinia* T3SS to inject the Yops effector proteins into host cells via the Ysc injectisome [27,43]. The V antigen regulates the Yops, is a terminal component of the infectosome and is essential to the process of host cell contact and translocation of the Yops into the cells [66,67,68,69,70].

The *pgm* locus includes several putative virulence factors and most notably the high-pathogenicity island genes essential for iron acquisition [54,71]. Inactivation of *pla,* on pPst, leads to the loss of plasminogen activator, an enzyme required for *Y. pestis* systemic dissemination after SC or aerosol exposure [44,55,72,73]. Despite its significant protective efficacy in this study, the CO92 *pgm* pPst-strain has potential weaknesses. Strains harboring a deletion of the *pgm* locus and having no other defined attenuating mutation have been shown to have residual virulence in nonhuman primates, in mice injected with excess iron, and in humans with abnormally high levels of serum iron, due to hereditary hemochromatosis [2,29,74,75,76,77]; the latter causes iron storage disease and permits the restoration of virulence and lethality to *Y. pestis pgm-*vaccine strains [76]. While the double *pgm*- pPst- mutant has an improved safety profile, it does not produce several antigens which could potentially facilitate immunity against a wider range of *Y. pestis* strains. For instance, in some, but not all studies, plasminogen activator was shown to induce a protective immune response and/or serve as a surrogate marker of infection [73,78,79,80]. Furthermore, the 102 kb *pgm* locus encodes several potential immunogens that may contribute to a protective immune response [53,54,81,82,83,84].

The vaccine efficacy findings of this study support either or both conclusions: (1) As shown in Table 3, Table 5, Table 6 and Table 7, these data strongly suggest that immune responses to the F1 capsule play an essential role in protection against the encapsulated *Y. pestis* strain CO92. Two vaccinations with the C12 Δ*yscN* mutant produced substantial levels of antibody to both of the whole bacterial antigens tested, but the vaccine failed to protect any animals against exposure to aerosolized CO92 and protected only 40% against SC challenge (Table 6). Obviously, in the *Y. pestis* C12 background, this strain did not induce anti-F1 antibody production. (2) It had been predicted that the absence of capsule would have theoretically presented multiple new surface antigens to the immune system. However, the C12 Δ*yscN* mutant may have failed to replicate well enough post-vaccination to induce an adequate immune response, especially after a single administration. As evidenced by the cytokine profiles obtained from splenocyte stimulation assays, the mice receiving only vaccine doses consisting of nonencapsulated strain had an altered immune response compared to mice receiving vaccine doses of an encapsulated strain (e.g., expression levels of Il-5, IL-13, and IL-9). Poor infectivity could have resulted from its inability to secrete the T3SS effector proteins, and especially the V protein, an essential virulence factor [27]; the absence of the anti-phagocytic activity of capsule in this mutant may have also played a role. These data clearly indicate the importance of the F1 antigen when protecting mice from CO92 challenge.

None of the Δy*scN* mutant-containing vaccines elicited anti-V antibody as expected, since the Δ*yscN* mutation impairs secretion of the Yops [27]. However, sera from mice vaccinated with a single dose of CO92 *pgm*-pPst-also induced little antibody response to the V antigen (Table 7). Interestingly, this lack of anti-V antibody stimulation has been reported for live plague vaccines by other investigators, to include data obtained by human vaccine subjects [32,58,59,60,61,62,63]. This finding possibly implies that anti-V antibody is not essential for the protection against encapsulated *Y. pestis*, although it appears to play a role in protection against both capsule-negative and capsule-positive strains by subunit vaccines (i.e., V protein alone or F1 and V combination vaccines) [8,85,86,87,88,89]. The exact role of V and other surface-exposed antigens in protection against virulent nonencapsulated *Y. pestis* strains remains to be clarified. One of our current efforts explores the role of antibodies to non-capsule surface components, such as the LPS, as described by Wang et al. [90], or to released antigens in addition to V in protective efficacy.

The CO92 *pgm*-pPst-, and to a lesser extent Δ*yscN* vaccine strains, elicited a cell-mediated as well as humoral immune response, as evidenced by the elevated levels of IFN-γ and IL-2 after F1-V stimulation. Furthermore, Th1-related chemokines MIP-1α (CCL3), MIP-1β (CCL4), and IP-10 (CXCL10) were also elevated [91]. The induction of Th2-like cytokines IL-4, IL-5, and IL-13 was also more pronounced in CO92 *pgm*-pPst-vaccinated groups, along with Th2 related chemokines MIP-2 (CXCL2) and MCP-3 (CCL7) [92,93]. The upregulation of Th17-related cytokines IL-17A along with IL-22 in those vaccine groups may synergize and enhance granulopoiesis, promote mucosal immunity through enhancement in antimicrobial peptides, and enhance neutrophil recruitment [25,94,95,96,97,98]. The presence of F1 in CO92 vaccine strains may be critical for promoting a stronger IL-17A response since all double dose CO92 vaccinated groups expressed higher levels of IL-17A than C12 vaccinated groups after stimulation with F1-V. Furthermore, GM-CSF, which drives the polarization of M1 pro-inflammatory macrophages, was also upregulated in Combo ×2 and CO92 *pgm*-pPst-vaccines [99].

Stimulation with whole cell antigens (CO92 or C12) enhanced the stimulation of almost all reported cytokines in the double dose vaccine groups relative to the single dose vaccine regiments. The Combo ×2 vaccinated group had an overall stronger cytokine response relative to all other vaccinated groups. The levels of IL-2 and IL-3 were significantly higher in the Combo ×2 vaccinated group after stimulation with CO92, while the levels of IL-3 and IL-4 were significantly higher in the Combo ×2 vaccinated group after stimulation with C12. IL-2 plays a major role in enhancing T cell immunity by means of CD4+ and CD8+ T cell activation, proliferation, and Foxp3 + regulatory T cell homeostasis [100,101]. IL-3 also plays a role in proliferation and survival. Dendritic cells that undergo maturation in the presence of IL-3 promote the expansion of Th2-like CD4+ T cells that in turn express more IL-4 and IL-5 but less IFN-γ [102]. Furthermore, the canonical Th2 associated cytokines, IL-4, IL-5, and IL-13, are induced to a greater level in the Combo ×2 vaccine group relative to the other groups. The recently identified group 2 innate lymphoid cells (ILC2) are non-T/non-B lymphoid-like cell group with no antigen-specific receptors on their surface appear to be major producers of IL-5, IL-9, and IL-13 [103]. IL-9 and IL-13 are involved in lung inflammation, mucus production by goblet cells, and augmentation of a Th2 immune response [104].

Capsule-producing vaccine strains (e.g., CO92 derived strains) conferred greater mouse survival post challenge, an enhanced antibody response, and stronger cytokine response relative to capsule negative C12 vaccine strains. The immune response induced by the encapsulated CO92 vaccine strains may enhance *Y. pestis* opsonization by alveolar macrophages in the lung mucosa after exposure to aerosolized virulent *Y. pestis*, reduce M cell traversal by *Y. pestis* thereby limiting bacterial dissemination, and enhance both Th17 and Th2 mucosal immune responses [105,106,107,108,109,110]. In the absence of rapid bacterial clearance post-challenge, a greater neutrophil influx that is ineffective at clearing *Y. pestis* bacteria could result in excessive inflammation and contribute to lung injury [111,112,113]. Nevertheless, even in the absence of a robust capsule and secretion of Yops effector proteins, a single dose of C12 Δy*scN* vaccine was able to confer 30% protection. This may demonstrate the existence of other protective antigens that may be critical at fortifying and expanding the currently recognized CO92 derived F1 and V mediated protection. Inclusion of additional booster vaccinations may increase the magnitude and longevity of the immune response. However, solely increasing the time interval between boosters without increasing the number of vaccinations may result in higher peak titers and increased levels of antibodies [114,115]. The highly repetitive F1 capsule polymers probably induced increased numbers of B cells, both long lived plasma and memory B cells, through activation by cross-linking multiple surface immunoglobulin molecules on the reactive B cell. Furthermore, due to the complex nature of the whole cell antigen, it is also able to recruit the T cell compartment, especially T follicular helper cells, for proper T cell dependent B cell activation that is critical for induction of long-lasting humoral immunity. Unfortunately, aside from F1, there appear to be no other polymeric immunogenic antigens that have been identified. Due to the highly attenuated nature of the C12 Δy*scN* mutants, the vaccine dose might have to be increased substantially to reach the antigenic threshold to induce more germinal center reactions to elicit a long-lasting protective antibody response [116,117].

CC and CXC chemokines are potent polymorphonuclear leukocytes (PMN) and neutrophil attractants that are involved in host defense against extracellular pathogens [118,119]. The pathogenesis of pulmonary plague involves both intracellular infection and systemic spread through the bloodstream [12]. MIP-1α deficiency results in poor neutrophilic infiltration to the infectious foci and lower levels of banded neutrophils [120]. In mice, the chemokines macrophage inflammatory protein-2 (MIP-2) and KC are major parts of the CXC family and are considered likely functional homologues of human IL-8 [121]. The secretion of these chemokines induces extravascular migration of neutrophils to sites of infection and the activation of clearance mechanisms; the latter appeared to be of major importance in a murine model of pneumonic plague [122]. The CXC chemokines are also produced in response to apoptosis or host cell damage, by signaling predominantly through CXC receptor 2 (CXCR2) and resulting in infiltration of PMNs to injured tissue to clean up dead cells [123], such as that which occurs in the necrotic bronchopneumonia of plague [122]. Furthermore, in addition to leukocyte recruitment, IP-10 can exert direct antimicrobial effect through membrane depolarization [124,125].

The absence of capsule in the C12 vaccine strain potentially exposed more antigens to the immune system than was the case with the F1-positive strain. F1 is known to induce primarily a T-cell independent humoral immunity [32,126,127], except perhaps when subunit antigens are delivered by a mucosal route, such as orally or intranasally [33,128]. Furthermore, the efficacy and ELISA data implied that the antibody response to F1 played a more significant role in protection, as described above.

Nevertheless, there is ample evidence that CMI contributes to vaccine responses to *Y. pestis*; CMI should continue to be evaluated in ongoing tests of vaccine efficacy against a larger range of *Y. pestis* strains and variants, such as strain C12. T-cell derived cytokines (especially TNF-α and IFN-γ) are reported to induce the antimicrobial functions of macrophages, such as reactive oxygen and nitrogen intermediates, and help them to combat infection by facultative intracellular pathogens such as *Y. pestis* [21,24,25,26,129,130]. Moreover, it is thought that antibodies and cellular responses both contribute to protection against plague independently; it was shown that cytokine responses (i.e., TNF-α and IFN-γ) conferred significant protection, even in the absence of a protective antibody [23,25,26]. Thus, it was argued that both arms of the immune system are necessary in protection, as confirmed in numerous studies [21,22,26,131,132]. The cytokine profile observed for the Δ*yscN* mutant vaccines was not identical to that described above; however, the cytokines/chemokines induced by such mutants might be expected to differ from those elicited by strains with a wild type functional *yscN* gene (and TTSS-encoded proteins).

## 5. Conclusions

In summary, our data contribute to the literature supporting the feasibility of live plague vaccines [3,27,28,30,31,32,33,47,48,58,61,63,79,90]. Novel candidate attenuated strains of *Y. pestis* were identified, which are capable of full protection against bubonic and pneumonic plague caused by the virulent CO92 strain of *Y. pestis*. The findings in this investigation encourage continued efforts to develop live vaccines which are optimally protective against lethal plague caused by a wide range of virulent strains of *Y. pestis.*

## Figures and Tables

**Table 1:** Primers used for generation of Δ*psp* mutants of *Y. pestis*.

*pspC* Primers for Mutant Construction and Screening	
pspCI-*Xho*I	CCGCTCGAGGGAACCCGCTTAACACCCAGTAG
pcpCI-*Bam*HI	CGGGATCCCAGTTTGAACGCCGTATTGACCAT
pspCI-lower-del	GTAGTTTTGTAGAAAATTCAACCG
pspCI-upper-del	TTATGACTGTCTCCAGTTAGGGTG
pspCII-lower-del	CATTATGACTGTCTCCAGTTAGGGTG
pcpCII-upper-del	TAGTTTTGTAGAAAATTCAACCG
***pspA* Primers for Mutant Construction and Screening**	
clone pspA 3′	CGGCAGGGATTAAACAGAGAAAAC
clone pspA 5′	AACGCGGGCAGATTATCATTGGTG
delete pspA 3′	TAGTTAATTTTCCGTATTTATTAG
delete pspA 5′	CATAATTTACGTCCCCTTTGACT
screen pspA 3′	GCGCGTGTAGGGGCAGGATT
screen pspA 5′	ATAAACCGAACGCTCTACCACATTT
**Screening for pCD1**	
lcrV-1	AGGGTGGAACAACTTACTG
lcrV-2	GTGCCACTACTAGACAGATGC
**Screening for pMT**	
Ymt-5′	TTTCGGCCAATCTCCAACAGTA
Ymt-3′	TCCGACCGCCCACATCA
CapAG-5′	AAAAATCAGTTCCGTTATCG
CapAG-3′	CTGCCCGTAGCCAAGAC
**Screening for pPst**	
Pla-5′	TGGCTTCCGGGTCAGGTA
Pla-3′	AGCCGGATGTCTTCTCACG

**Table 2:** Virulence attenuation in mice of candidate *Y. pestis* live plague strains.

*Y. pestis*		LD_50_ by Route (no. CFU) ^a^			
Strain ^b^	Characteristics ^c^	SC	Aerosol	Intranasal	References ^d^
CO92	wild type	1−2 × 10^0 f^	6.8 × 10^4 f^	1.4 × 10^3 f^	41, 50
CO92 *pgm*- pPst-	*pgm*- (102 kb deleted by serial passage on congo red agar), pPst^-^ (cured by serial passage on agar 4 °C)	>1.0 × 10^8 f^	nd ^e^	nd	6, 42, 54
CO92 ∆*tat*A	in frame deletion of *tat*A	1.5 × 10^7^	>9.4 × 10^5^	2.4 × 10^3^	38
CO92 ∆*yscN*	in frame deletion of *yscN*	>3.2 × 10^7^	nd	nd	27, 43
CO92 ∆*pspA*	in frame deletion of *pspA*	6 × 10^2^	>1.0 × 10^6^	nd	Current study
CO92 ∆*pspCI*	out of frame (potentially polar) deletion of *pspC*	>2.1 × 10^5^	>4.5 × 10^6^	nd	Current study
CO92 ∆*pcpCII*	in frame deletion of *pcpC*	3.1 × 10^3^	>1.9 × 10^6^	nd	Current study
C12	F1^-^ CO92; site directed mutagenesis of *caf1A*	9 × 10^0 f^	7.7 × 10^4 f^	nd	7
C12 ∆*yscN*	in frame deletion of *yscN*, *caf1A*	>2.0 × 10^7 f^	nd	nd	Current study
KIM6+	wild type	<1.0 × 10^1^	nd	~1.0 × 10^2^	45
KIM6+ χ10030/ pCD1Ap1	*lpxL*, *crp* (See references)	>1.0 × 10^8^	nd	>1.0 × 10^6^	46–48

^a^ The LD_50_s were those determined previously in Swiss Webster mice, except as indicated in footnote f. ^b^ All mutants were derived from the wild type *Y. pestis* CO92 strain or its F1-negative C12 derivative (*caf1A*), except for χ10030/pCD1Ap1, from the *pgm*+ strain KIM6+. ^c^ Includes attenuating mutations as described in the References cited. ^d^ Cited in References section, except for the CO92 *pspC* and C12 ∆*yscN* mutants, which are described herein. ^e^ nd—not done. ^f^ LD_50_ determined in BALB/c mice. > # indicates highest dose tested in BALB/c mice.

**Table 3:** Protection of BALB/c mice with *Y. pestis* vaccine strains against virulent *Y. pestis.*

Vaccine				Challenge	
Strain ^a^	Dose (no. CFU) ^b^	No. mice ^c^	Survival (%) ^d^	Route ^e^	Survival (%)
KIM 6+ χ10030/pCD1Ap ^f^	4.6 × 10^6^	10	0	SC	nd ^g^
	7.2 × 10^5^	20	35	SC	nd
CO92 *pgm*-pPst-	6.2 × 10^6^	10	100	SC	100
CO92 ∆*yscN*	7.2 × 10^6^	10	100	SC	100
C12 ∆*yscN*	1.7 × 10^7^	10	100	SC	30
CO92 mutants ^g^	0.5–1.2 × 10^7^	10	0	nd	nd
Kphos	NA	10	100	SC	0
CO92 *pgm*-pPst-	7.6 × 10^6^	10	100	AERO	100
CO92 ∆*yscN*	1.0 × 10^7^	10	90	AERO	100
C12 ∆*yscN*	1.7 × 10^7^	10	100	AERO	0
Kphos	NA	10	100	AERO	0

^a^ Includes mutations which inactivate genes that attenuate virulence. An additional cohort of 10 mice per group were euthanized prior to challenge to collect spleens and sera for immunological tests. ^b^ The target dose for all strains was 1 × 10^7^ CFU, as used for χ10030/pCD1Ap (3, 45); except for the group receiving the lower dose of χ10030/pCD1Ap. ^c^ *n* = 10 mice/group, except 20 for the lower dose χ10030/pCD1Ap group. ^d^ The number of mice (%) which survived exposure to the vaccine strains. ^e^ Mice were challenged subcutaneously (SC) with *Y. pestis* CO92, 235 LD_50_s (376 CFU) for all except 206 LD_50_s (329 CFU) for C12 ∆*yscN* group. Mice were aerosol (AERO)-challenged with 8 LD_50_s (5.18 × 10^5^ CFU) of wt CO92; the C12 ∆*yscN* vaccinees received 22 LD_50_s (1.52 × 10^6^ CFU) of wt CO92. All mice were challenged 28 days after vaccination. ^f^ Derived from *pgm*+ wild type *Y. pestis* stain KIM6+. Strain χ10030/pCD1Ap1 expresses an *araP*- controlled *crp* gene activator, and *lpxL*, an acylacetylase which produces a TLR4-reactive LPS variant. ^g^ Four other CO92 mutants were tested (single mutations, *tatA*, *pspA*, *pspC*I, or *pspC*II). All mice succumbed post vaccination, nd—not done.

**Table 4:** Protection of CD-1 mice with live *Y. pestis* vaccine strains.

Vaccine				Challenge	
Strain ^a^	Dose (no. CFU) ^b^	No. Mice ^c^	Survival (%) ^d^	Route ^e^	Survival (%) ^e^
CO92 *pgm*-pPst-	8.2 × 10^6^	10	100	SC	100
CO92 *∆yscN*	9.4 × 10^6^	10	100	SC	100
Kphos	NA	10	100	SC	30
CO92 *pgm*-pPst-	7.6 × 10^6^	10	100	AERO	60
CO92 *∆yscN*	1.0 × 10^7^	10	100	AERO	20
Kphos	NA	10	100	AERO	0

^a^ Includes mutations which inactivate genes that attenuate virulence. An additional cohort of 10 mice per group were euthanized prior to challenge to collect spleens and sera for immunological tests. ^b^ The target dose for all strains was 1 × 10^7^ CFU. ^c^ The numbers of mice (%) which survived exposure to the live vaccine strains. ^d^ Mice were challenged by the subcutaneous (SC) route with 478 LD_50_s of *Y. pestis* CO92 (2.5 × 10^4^ CFU) or were challenged by the aerosol (AERO) route with 26 LD_50_s of *Y. pestis* CO92 (8.71 × 10^5^ CFU). ^e^ Mice exposed to aerosolized bacteria were challenged 28 days after vaccination and mice infected via the SC route were challenged 30 days after vaccination.

**Table 5:** Humoral immune responses elicited by one dose of *Y. pestis* vaccine strains.

		IgG ^c^	
Vaccine ^a,b^	Capture Antigen	Geo Mean	(GSE)
Kphos ^a^*	CO92	50	(1.08)
	C12	50	(1.08)
	F1-V	50	(1.08)
CO92 *pgm*-pPst-	CO92	5572	(1.45)
	C12	3592	(1.51)
	F1-V	160,000	(1.16)
CO92 Δ*yscN*	CO92	348	(1.38)
	C12	470	(1.41)
	F1-V	89,797	(1.24)
C12 ΔyscN ^a^**	CO92	1819	(1.53)
	C12	2604	(1.79)
	F1-V	61	(1.17)
CO92 Δ*yscN* + C12 Δ*yscN*	CO92	1008	(1.68)
	C12	1158	(1.67)
	F1-V	40,317	(1.71)

^a^*n* = 10 for each group of mice, except * *n* = 8; ** *n* = 9. ^b^ Single SC vaccination. ^c^ 30 °C antigens. Reported as geometric mean (Geo Mean) with geometric standard error (GSE).

**Table 6:** Protection of BALB/c mice with live *Y. pestis* vaccine strains versus virulent challenge.

Vaccine Dose (no. CFU) ^b^					Challenge ^c^	
Strain ^a^	1st	2nd	No. Mice	Survival (%)	Route	Survival (%)
CO92 *∆yscN* × 2 ^d^	1.03 × 10^7^	0.85 × 10^7^	10	100	SC	100
C12 *∆yscN* × 2	1.27 × 10^7^	0.93 × 10^7^	10	100	SC	40
Combo × 2	0.95 × 10^7^	0.93 × 10^7^	10	100	SC	100
CO92 *∆yscN*	NA	0.85 × 10^7^	10	100	SC	100
C12 *∆yscN*	NA	0.93 × 10^7^	10	100	SC	30
Combo	NA	0.93 × 10^7^	10	100	SC	90
Kphos	NA	NA	10	100	SC	0
CO92 *∆yscN* × 2	1.03 × 10^7^	0.85 × 10^7^	10	100	AERO	100
C12 *∆yscN* × 2	1.27 × 10^7^	0.93 × 10^7^	10	100	AERO	0
Combo × 2	0.95 × 10^7^	0.93 × 10^7^	10	100	AERO	90
Kphos	NA	NA	10	100	AERO	0

^a^ Includes mutations which inactivate genes that attenuate virulence. An additional cohort of 10 mice per group were euthanized prior to challenge to collect spleens and sera for immunological tests. ^b^ The target dose for all strains was 1 × 10^7^ CFU.^c^ Mice were challenged subcutaneously (SC) with *Y. pestis* CO92, 316 LD_50_s (505 CFU). Mice were aerosol (AERO) challenged with *Y. pestis* CO92, 7 LD_50_s (4.78 × 10^5^ CFU). All mice were challenged 28 days after vaccination.^d^ ×2: These groups received an initial vaccination followed by booster vaccine 23 days later.

**Table 7:** Humoral responses to F1 and V antigens by *Y. pestis* vaccine strains.

		IgG ^a^	
Vaccine ^b,c^	Capture Antigen	Geo Mean (GSE)	
Kphos	F1	50	(1)
	V	50	(1)
CO92 *pgm*-pPst-	F1	312,691	(1.18)
	V	235	(1.42)
CO92 Δ*yscN*	F1	23,829	(1.40)
	V	51	(1.04)
C12 ΔyscN ^b^*	F1	50	(1)
	V	50	(1)
CO92 Δ*yscN* + C12 Δ*yscN*	F1	6400	(1.45)
	V	50	(1)
CO92 *pgm*- pPst- ×2 ^b^**	F1	926,252	(1.17)
	V	729	(2.22)
CO92 Δ*yscN* × 2	F1	113,137	(1.27)
	V	120	(1.32)
C12 Δ*yscN* × 2 ^b^*	F1	50	(1)
	V	59	(1.12)
CO92 Δ*yscN* + C12 Δ*yscN* × 2	F1	118,488	(1.27)
	V	71	(1.13)

^a^ 30 °C antigens. Antibody titers are the geometric mean (Geo Mean) with geometric standard error (GSE). ^b^ *n* = 10 for each group of mice, except * *n* = 9. ** *n* = 5; mice used for immunological analyses (not challenged with virulent plague) and these mice received the booster vaccine 28 days after the primary vaccination. ^c^ Single SC vaccination. Double SC vaccination if notated ×2.

**Table 8:** Humoral immune responses elicited by vaccines to antigens prepared at 30 °C.

Vaccine ^b,c^	Capture Antigen	IgG ^a^		IgG1 ^a^		IgG2a ^a^		Ratio IgG2a/IgG1
		Geo Mean (GSE)		Geo Mean (GSE)		Geo Mean (GSE)		
Kphos	CO92	50	(1)	50	(1)	50	(1)	
	C12	50	(1)	50	(1)	50	(1)	
CO92 *pgm*-pPst-	CO92	5572	(1.45)	9902	(1.54) ^b,^*	312	(1.68) ^b,^*	0.03
	C12	3592	(1.51)	16,977	(1.40) ^b,^*	352	(1.54) *	0.02
CO92 Δ*yscN*	CO92	746	(1.68)	1213	(1.82)	118	(1.54)	0.10
	C12	650	(1.64)	1477	(1.77)	132	(1.59)	0.09
C12 Δ*yscN*	CO92	504	(1.68)	1241	(1.78)	83	(1.34)	0.07
	C12	540	(1.77)	1080	(1.69)	107	(1.42)	0.10
CO92 Δ*yscN* + C12 Δ*yscN*	CO92	276	(1.62)	696	(1.56)	89	(1.35)	0.13
	C12	449	(1.62)	504	(1.38)	98	(1.37)	0.19
CO92 *pgm*-pPst- ×2 ^b,^**	CO92	320,000	(1.21)	485,029	(1.19)	12,222	(1.37)	0.03
	C12	320,000	(1.26)	884,424	(1.54)	16,127	(1.21)	0.02
CO92 Δ*yscN* ×2	CO92	19,097	(1.25)	61,110	(1.28)	2202	(1.97)	0.04
	C12	19,543	(1.32)	44,221	(1.33)	2416	(1.91)	0.05
C12 Δ*yscN* ×2 ^b,^*	CO92	37,998	(1.41)	45,920	(1.96)	1553	(2.22)	0.03
	C12	22,202	(1.46)	36,378	(2.11)	1142	(2.05)	0.03
CO92 Δ*yscN* + C12 Δ*yscN* ×2	CO92	27,007	(1.33)	97,006	(1.33)	1711	(1.90)	0.02
	C12	29,622	(1.26)	122,204	(1.43)	2106	(1.70)	0.02

^a^ 30 °C antigens. Antibody titers are shown as the geometric mean (Geo Mean) with geometric standard error (GSE). ^b^ *n* = 10 for each group of mice, except * *n* = 9. ** *n* = 5; mice used for immunological analyses (not challenged with virulent plague) and these mice received the booster vaccine 28 days after the primary vaccination. ^c^ Single SC vaccination. Double SC vaccination if notated ×2.

**Table 9:** Humoral immune responses elicited by vaccines to antigens prepared with change in temperature (30–37 °C).

Vaccine ^b,c^	Capture Antigen (Temp. Shift)	IgG ^a^		IgG1 ^a^		IgG2a ^a^		Ratio IgG2a/IgG1
		Geo Mean (GSE)		Geo Mean (GSE)		Geo Mean (GSE)		
Kphos	CO92	51	(1)	50	(1)	50	(1)	
	C12	54	(1)	50	(1)	50	(1)	
CO92 *pgm*-pPst-	CO92	42,559	(1.23)	144,009	(1.19)	1754	(1.36)	0.01
	C12	9,263	(1.34)	23,886	(1.33)	558	(1.38) ^b^*	0.02
CO92 Δ*yscN*	CO92	17,688	(1.28)	20,319	(1.36)	1213	(1.52)	0.06
	C12	1,459	(1.67)	1925	(1.70)	235	(1.62)	0.12
C12 Δ*yscN* ^b,^*	CO92	933	(1.83)	1034	(1.77)	205	(1.67)	0.20
	C12	864	(1.80)	1444	(1.89)	143	(1.50)	0.10
CO92 Δ*yscN* + C12 Δ*yscN*	CO92	8844	(1.48)	12,498	(1.64)	439	(1.82)	0.04
	C12	579	(1.58)	566	(1.71)	219	(1.57)	0.39 ^d^
CO92 *pgm*-pPst- ×2 ^b,^**	CO92	403,175	(1.21)	583,502	(1.37)	20,319	(1.34)	0.03
	C12	115,782	(1.27)	305,549	(1.43)	3850	(1.58)	0.01
CO92 Δ*yscN* ×2	CO92	44,221	(1.24)	116,700	(1.18)	11,633	(1.47)	0.10
	C12	32,748	(1.33)	34,324	(1.47)	2154	(1.87)	0.06
C12 Δ*yscN* ×2 ^b,^*	CO92	52,072	(2.06)	106,315	(1.58)	3813	(2.04)	0.04
	C12	27,284	(1.92)	23,557	(1.73)	1367	(2.23)	0.06
CO92 Δ*yscN* + C12 Δ*yscN* ×2	CO92	113,945	(1.44)	272,860	(1.33)	8412	(2.08)	0.03
	C12	80,508	(1.43)	34,243	(1.4)	1452	(2.32)	0.04

^a^ Temperature shifted antigens. Antibody titers are shown as geometric mean (Geo Mean) with geometric standard error (GSE). ^b^ *n* = 10 for each group of mice, except * *n* = 9. ** *n* = 5; mice used for immunological analyses and (not challenged with virulent plague) these mice received the booster vaccine 28 days after the primary vaccination. ^c^ Single SC vaccination. Double SC vaccination if notated×2. ^d^ We postulate that the exaggerated IgG2a/IgG1 ratio is an artifact due to the low titers.

## Data Availability

The data presented in this study are available on request from the corresponding author.

## Supplementary Materials

The following is available online at https://www.mdpi.com/2076-393X/9/2/161/s1, Figure S1: The PSP proteins are necessary for plague by either bubonic or pneumonic infection. Groups of Swiss Webster mice were challenged by either the subcutaneous or whole body aerosol route, as indicated, with the designated CO92 mutant strain. The calculated LD_50_ values are included in Table 2.
