# Supplementary material for: Protection Elicited by Attenuated Live Yersinia pestis Vaccine Strains against Lethal Infection with Virulent Y. pestis
Source: Vaccines (Basel). 2021 Feb 16;9(2):161. doi: 10.3390/vaccines9020161 (PMC7920443; doi:10.3390/vaccines9020161)
Supplement: Supplementary file 1 [file vaccines-09-00161-s001.pdf]

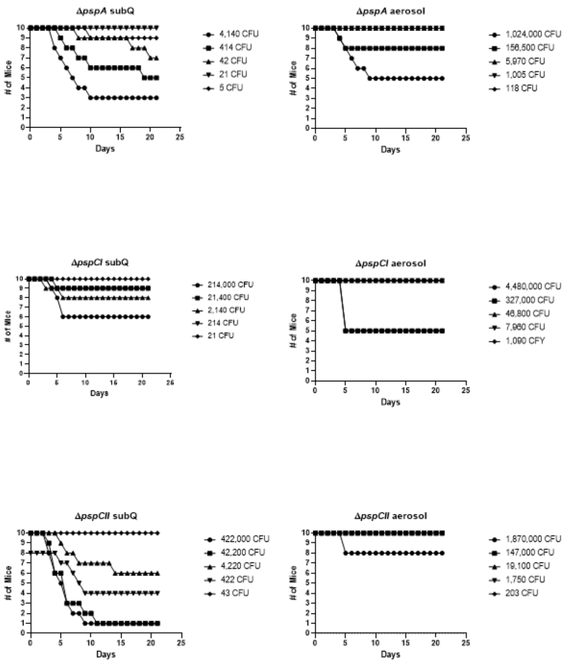


**Figure S1.** The PSP proteins are necessary for plague by either bubonic or pneumonic infection. Groups of Swiss Webster mice were challenged by either the subcutaneous or whole body aerosol route, as indicated, with the designated CO92 mutant strain. The calculated $LD_{50}$ values are included in Table 2.
